# Presence of Virulence-Associated Genes and Ability to Form Biofilm among Clinical Isolates of *Escherichia coli* Causing Urinary Infection in Domestic Animals

**DOI:** 10.4236/aim.2015.58059

**Published:** 2015-07-30

**Authors:** Cherise Hill, Marianne Pan, Lmar Babrak, Lia Danelishvili, Helio De Morais, Luiz E. Bermudez

**Affiliations:** 1 Department of Biomedical Sciences, College of Veterinary Medicine, Oregon State University, Corvallis, USA; 2 Department of Microbiology, College of Science, Oregon State University, Corvallis, USA; 3 Department of Clinical Sciences, College of Veterinary Medicine, Oregon State University, Corvallis, USA

**Keywords:** *E. coli*, Pathogenesis-Related Genes, Biofilm, Urinary Tract Infection, Animals, UPEC

## Abstract

**Background:**

Urinary tract infection caused by *Escherichia coli* is a frequently observed condition both in humans and animals. Uropathogenic *E. coli* (UPEC) has been shown to have a pathogenicity island that enables them to infect the urinary tract. Because there is little information about the presence of UPEC-associated virulent genes in animal isolates this work was carried out with the intent to enhance the understanding about the strains of *E.coli* that cause infections in animals.

**Results:**

We screened 21 *E. coli* strains isolated causing urinary tract infection in domestic animals. Primers were designed to amplify urinary infection-associated genes. Nine genes, *pap*A, *tcp*C*, fyu*A, *tpb*A, *Lma, hyl*A, *pic*U, *ton*B, and *flic*C were then amplified and sequenced. Different from the human isolate CFT073, all the animals *E. coli* lack some of the pathogenesis-associated genes. Genes encoding for proteins used to scavenge iron appear not to be so necessary during animal infections as they are in human infection. In further investigation of phenotypic properties, it was observed that animal UPECs have significantly more impaired ability to form biofilms than human UPEC strain.

**Conclusions:**

This study identified significant differences between human and animal UPECs. This may have its roots in the fact that it is difficult to determine if an animal has symptoms. Future studies will focus on some of the observations.

## 1. Introduction

The urinary tract infection is a common location for bacterial infection in both humans and dogs [1] [2]. Many virulence factors are associated with the ability of the uropathogenic *Escherichia coli* (UPEC) to cause disease in humans [3]-[5]. UPEC normally inhabits the intestinal tract and transition by contaminating the uroepithelial mucosa of the urethra. The bacteria then need to attach to the epithelial surface, a step depending on the presence of virulence factors, such as P adhesin and Type 1 fimbria [6] [7]. Once attached, the microorganism can migrate upwards towards the bladder and kidneys. The importance of additional virulence factors for the subsequent phase of infection has been demonstrated [8]. For example, secreted toxins, as well as bacterial capsule contribute to the ability to colonize. In addition, the lower urinary tract contains bactericidal proteins, such as defensin-3 and defensin-14 which have been shown to be bactericidal to *E. coli* [3], requiring the bacterium to have specific defense mechanisms.

There are several *E. coli* pathotypes, including EPEC, EHEC, ETEC, UTEC, and UPEC. What differentiates the majority of the pathotypes is the presence of unique pathogenicity islands, an acquired segment of foreign DNA, which allows the pathogen to adapt to specific environments [9] [10]. UPEC, therefore, has specific characteristics that allow infecting the urinary tract. Although many studies have been carried out in the identification of molecular signatures of UPEC human isolates, very little is known about urinary tract *E. coli* that infects dogs and other domestic animals. At the Oregon State University Veterinary Teaching Hospital, *E. coli* cases of urinary tract infection represent approximately 40% of the identified cases during the past 5 years. Infections in dogs were responsible for 89% of the UTI cases in small animals. Understanding the pathogenesis of the UPEC infection in domestic animals would be important since the risk factors associated with the infection may differ from the ones recognized in humans.

To begin to define the factors associated with dog, cat and other domestic animal’s urinary tract infection, we collected *E. coli* organisms isolated from the urine, both of hospitalized animals and from ambulatory animals, and investigated the presence of nine genes that have been associated with human disease. As positive control, we used the strain CFT073 known to express all of the genes. Because the ability to form biofilm is linked with successful urinary tract infection in hospitals, we also tested the animal strains for their ability to form biofilm in comparison to the strain CFT073.

## 2. Material and Methods

### 

#### Ethical Statement:

Animals were not used in this work. Bacteria isolated from urine were obtained from the microbiology laboratory. Information about the animals was collected from the clinician’s note. In the cases the charts were consulted the information regarding to the animal was not recorded. All the work was performed in compliance with the guidelines of the IACUC of Oregon State University.

### 2.1. *E. coli* Strains

*E. coli* CFT073 was obtained from ATCC (Manassas, VA). Twenty clinical isolates were obtained from dogs, cats, horses, llamas, alpacas and pigs. The strains were initially cultured on LB agar plates (Fisher Laboratories) as isolated colonies. The isolated colonies were then checked for purity. Genomic DNA was obtained as previously described [11].

### 2.2. Epidemiological Data

Surveillance of infectious diseases is conducted in the hospital on a monthly basis. Identified cases of urinary tract infection by microbiological culture undergo antibacterial susceptibility using standard techniques of antibacterial diffusion. Results are analyzed and if necessary control measures are implemented [11]. For this study, all the isolates were associated with symptoms such as difficulty to urinate, pain and fever. Four of the patients had urinalysis performed before culture.

### 2.3. DNA Amplification and Sequencing

DNA for nine genes associated with urinary infection in humans was amplified using the primers shown in Table 1. Amplification was carried out as follows: 95°C for 5 min, 95°C for 30 s, 56°C for 30 s, and 72°C for 1 min (35 cycles), 72°C for 5 min and 4°C (hold). The PCR products were then run in an agarose gel, bands were cut and submitted for sequencing at the OSU Center for Genomic Research and Biocomputing. The genes amplified, their location in the genome and putative function are shown in Table 2.

### 2.4. Ability to Form Biofilm

To verify if the *E. coli* strains would form biofilm and compare their biofilm with the biofilm of the strain CFT073, agar-cultured *E. coli* strains were added to 10 ml of LB broth, and incubated overnight under constant agitation. Then 1 ml of the suspension was collected and the concentration adjusted to approximately 10^7^ bacteria using the McFarland 1 standard. One hundred ml of the suspension was inoculated in four wells of a 96-well plate (Corning, Corning, NY) and incubated at 37°C for 4, 12, 24 and 72 h. At each of the time points, the supernatant was discarded, the wells washed once with HBSS and 100 μL of stain crystal violet (Sigma Co., St. Louis, MO) was added to each well for 15 min. The crystal violet was discarded and the amount of the dye retained in the biofilm was solubized in 100% ethanol. The plate then was read in a spectrophotometer using a 600 nm filter. As controls, empty wells and biofilm formed by CFT073 were used. The results were normalized with the data obtained from empty wells and the absorbance value was compared between controls and the experimental groups.

## 3. Results

### 3.1. Urinary Tract Infection Diagnosed in the College of Veterinary Medicine

Between June 2006 and June 2013, 360 cases of microbiologically confirmed urinary tract infection were diagnosed at the microbiology laboratory of the teaching hospital at Oregon State University. Fifty-five percent of the cases were from canine, 9% from feline, 10% equine, 9% camelid and 9% porcine. Eight percent of the cases were from other additional species. *E. coli* was the most common bacterium isolated in every year but except one.

### 3.2. Presence of Virulence Genes Associated with UTI

The presence of the genes *pap*A, *Lma, ton*B, *fyu*A, *tpb*A, *pic*U, *hyl*A, *fli*C and *tcp*C were examined in the genomes of 21 strains of UPEC isolated from animals in the veterinary hospital. All strains contain *pap*A, encoding for a pilus responsible for the binding to epithelial cells and all but one of the strains contained *tcp*C. The majority of the strains contained *Lma* and *fyu*A genes, both iron acquisition proteins. The rest of the genes had varying representation with no clear pattern. As expected, the CFT073 strain contains all genes. Table 3 shows the distribution of the genes among the strains tested.

### 3.3. Biofilm Formation

**Figure 1.**
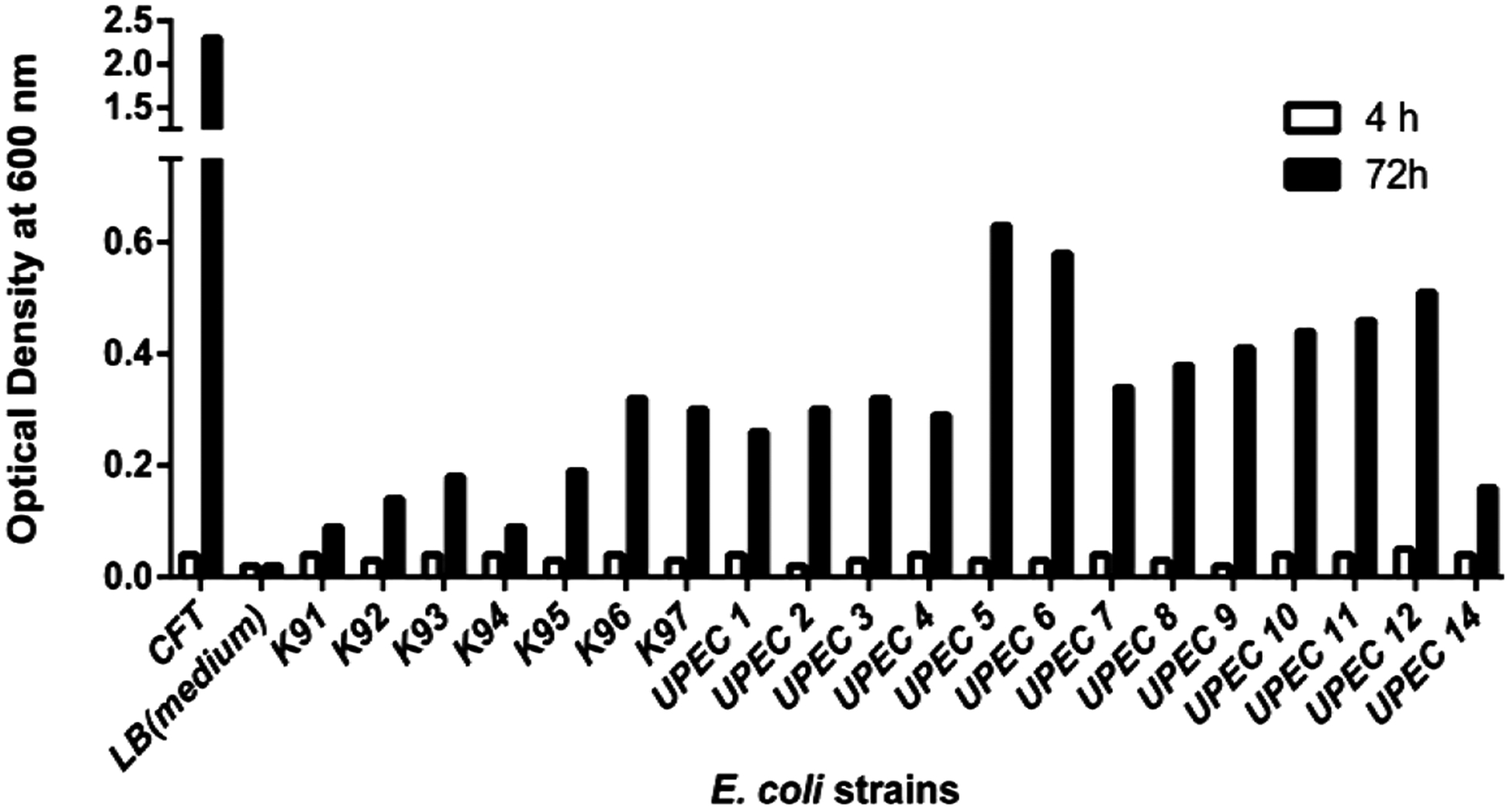
Biofilm formation by strains of *E. coli* isolated causing urinary tract infection in animals.

The ability to form biofilm is very important for the establishment of urinary tract infection in humans [6] [12]. We explored the question about the importance to form biofilm in animal UTI. Twenty-one strains of *E. coli* were evaluated for the ability to form biofilm using the traditional method of biofilm formation on plastic surface. As observed in Figure 1, only 4 out of 21 strains were able to form biofilm with less mass and robust, whereas the majority of the clinical samples formed very small biofilms. After 72 h, the strain CFT073 formed significantly greater biofilm than any of the 21 animal derived strains. Samples with clinical history of recurrent UTIs were the UPEC 9 and UPEC 10. Sample 10 had a diagnosis of “drug-resistant *E. coli*”, and sample 10 had a prior history of UTIs as well as recurring UTIs throughout her treatment at the Veterinary Hospital. They also did not show significant biofilm formation which was unexpected (Figure 1).

## 4. Discussion

Urinary tract infection is a common condition in humans and animals. In animals, like in humans, the majority of the cases of urinary tract infection are due to contamination of the urinary tract with intestinal bacteria. *E. coli*, and enterococcus are the most common isolates causing infection [1] [12]. Bacteria have access to the urinary tract facilitated by the proximity between the anus and the urethra, or in some cases, by the presence of a plastic catheter in the urinary tract. The latter, although very common mechanism of infection in hospital-acquired infections in humans, is not as relevant for animals.

UPEC is capable of causing infection because it contains the necessary genetic tools. For example, the type I fimbria, flagellum and pili have been associated in many studies with the ability to infect the urinary tract [2] [6]. Genes encoding for proteins that successfully compete for iron in the urinary tract environment have been also demonstrated to be important for the fitness in the site [13] [14]. Recent studies have shown that additional genes, however, are necessary in *E. coli* to cause systemic infection [8], and those are entirely different from the genes associated with the capability to cause urinary tract infection.

Survey of our hospital microbiology laboratory determined that *E. coli* was the most frequent pathogen isolated causing urinary tract infection in animals, with an incidence of approximately 40% of the urinary infections. Then, we screened twenty-one randomized strains isolated from symptomatic animals for the presence of genes linked to the ability to cause human infection. The majority of genes are present in the pathogenicity island specific for strains of UPEC [4] [5].

All the strains examined expressed flagellar and pilin genes, confirming their importance in the establishment of the urinary tract infection. Only one of the strains of *E. coli* tested did not have *fli*C. The strain was obtained from a dog and had no specificcolony morphotypeas observed on agar plates. The *pap*A gene was very well conserved among the tested strains. Among the iron-sequestering proteins, *tpb*A is present in all of the strain tested while *Lma* and *ton*B were absent in many strains. In five of the isolates, all obtained from dog infection, *Lma* and *ton*B were absent, and only the PCR product for *tpb*A was amplified. If the iron requirements of UPECs infecting dogs is smaller than the need of UPEC infecting humans or other animals is currently unknown, but is a possibility to be considered. The absence of *hyl*A in almost 50% of the strains tested may indicate either that hemolysis is not a necessity to obtain iron from the environment or that the animal derived strains either do not depend as much as the human strains on iron, or iron is more available in the urinary tract of animals in contrast to the urinary tract of humans. The presence of *hyl*A is also associated with bacterial dissemination and the decreased frequency *hyl*A in animals, UPECs may have been selected for strains that do not contain *hyl*A.

The ability to form biofilm is crucial for catheter-related infections [1] [2]. Differently from the human isolate CFT073, all the 21 animals isolates were different in the ability to form biofilm. This observation may have important interpretations. Maybe, the small incidence of catheter-related infection in animals has allowed for infections by poor-biofilm forming *E. coli*. Alternatively, urinary infections in animals might be less dependent on biofilm formation than in humans. Since the genes encoding for proteins associated with biofilm formation in UPEC such as type I fimbria, flagellum and Ag43 [6] have been previously identified, future study may look for the expression of those genes in animal UPEC’s.

Finally, there is a possibility that the failure to detect several of the UPEC genes in many of the strains might be due to divergency sequence of the genes. Although possible, it is very unlikely for a couple of reasons. The genes selected are conserved gene and the sequences used for the PCR amplification were conserved sequences in the genes. Our study has a limitation, which is the fact that animals do not vocalize their complaints and most of the times, the owner report the possible symptoms and signs. Therefore, some of the studied animals may not have UPECs, but colonizing *E. coli*. The fact, however, that none of the 21 strains evaluated showed the complete number of genes, make us assume that animal-associated *E. coli* UPECs differ from human UPECs.

The obtained observation identify important questions related to the pathogenesis of UTI’s in animals that future investigations should address.

In summary, our study although limited because of the number of strains screened, nonetheless, has made some important observations. The differences in the genome between a model human UPEC strain and *E. coli* strains obtained from animals urinary infections appear to suggest important differences between both. Future investigations should address the raised questions.

## 5. Conclusion

This work determined that the animal strains of *E. coli* do not have all the virulence genes associated with the classical UPEC of humans. The reason for it is unknown at this point. Also, the majority of the strains tested were poor biofilm formers, in contrast with the standard UPEC strain from human infection. If the pressure to form biofilm in the animal urinary infection differs from the pressure/conditions that stimulate biofilm formation in human urinary tract infection caused by *E. coli*, the topic warrants additional investigation.

## Figures and Tables

**Table 1.** Primers used in this study.

Genes	Forward	Reverse
*pap*A	GGAGCGTCAACAACAGT	GCGGTAGCTATGGCAGTG
*tcp*C	TATATGCTTGGTGAATGT	AGCCAACTCTCTTGCTAT
*fyu*A	AATGCCCAGACTTCACA	TTCACATTCCCTATGCT
*tpb*A	TGCCAGCATTAGCGTCTT	GATAGCCCAGCGTCAC
*Lma*	CACTCTGTCATTATGCAT	GTGGAGTTCTGCAATGT
*hyl*A	AGCACACTACAGTCTGCA	CCTGCACCAATATTATC
*pic*U	CACGCAGTACCGTCTCAC	GTCAACAATCTGAACAAACG
*ton*B	CTCTCGTTCGATGAAGGATT	AGCGTAAATACTCAGCAGAG
*fli*C	CTTCTGGCTTGCGTATTAAC	CCTGAATTTTCATCGAACCG

**Table 2.** UTI associated genes, location in the genome and function.

Gene	Function	Genome location (CFT073)	Presence among strains
*pap*A	Pilus adhering to epithelial cell during infection	PAI-I	Present in all
*hly*A	Yersiniabactin receptor	PAI-I	Present in 12/21
*tpb*A	Iron acquisition cluster	PAI-III	Present in all
picU	Serine protease autotransporter	PAI-III	Present in 15/21
*pyu*A	Pesticin-receptor	PAI	Present in 19/21
*Lma*	Outer membrane receptor for iron		Present in 14/21
*Tcp*C	Hypothetical protein	PAI	Present in 4/21
tonB	Sichropolar		Present in 11/21
*fliC*	Flagellum		Present in 20/21

**Table 3.** Presence of UPEC genes (UTI-associated) in 21 strains and CFT073 strain.

Strain	*pap*A	*Lma*	*ton*B	fliC	*fyu*A	*pic*U	*hyl*A	*tcp*C	*tpb*A
**CFT073**	+	+	+	+	+	+	+	+	+
**K91**	+	+	+	+	+	+	+	+	+
**K92**	+	−	−	+	+	−	+	−	+
**K93**	+	−	−	+	+	−	+	−	+
**K94**	+	−	−	+	+	−	+	−	+
**K95**	+	−	−	+	+	+	−	−	+
**K96**	+	−	+	+	+	−	+	+	+
**K97**	+	−	+	+	−	+	−	−	+
**K98**	+	−	−	−	−	+	−	−	+
**1**	+	+	−	+	+	+	−	−	+
**2**	+	+	+	+	+	+	−	−	+
**3**	+	+	+	+	+	+	+	−	+
**4**	+	+	+	+	+	+	+	−	+
**5**	+	+	+	+	+	+	−	+	+
**6**	+	+	+	+	+	+	−	−	+
**7**	+	+	−	+	+	+	+	−	+
**8**	+	+	−	+	+	+	−	−	+
**9**	+	+	−	+	+	+	+	−	+
**10**	+	+	+	+	+	+	−	+	+
**11**	+	+	−	+	+	+	+	−	+
**12**	+	+	+	+	+	+	+	−	+
**14**	+	+	+	+	+	+	+	−	+
